# The Influence of Fluorination on Nano-Scale Phase Separation and Photovoltaic Performance of Small Molecular/PC_71_BM Blends

**DOI:** 10.3390/nano6040080

**Published:** 2016-04-22

**Authors:** Zhen Lu, Wen Liu, Jingjing Li, Tao Fang, Wanning Li, Jicheng Zhang, Feng Feng, Wenhua Li

**Affiliations:** 1 College of Chemistry and Environmental Engineering, ShanXi DaTong University, Datong 037009, China; luzhen0313@aliyun.com (Z.L.); liuwen19701021@163.com (W.L.); lijingjing8150@163.com (J.L.); liwanning2016@163.com (W.L.); 2 Beijing Key Laboratory of Energy Conversion and Storage Materials, College of Chemistry, Beijing Normal University, Beijing 100875, China; fangtao@bnu.edu.cn (T.F.); zhangjichengbnu@hotmail.com (J.Z.)

**Keywords:** small molecule, fluorinated phenyl (DFP) groups, organic solar cell, solution process, nanoscale phase separation

## Abstract

To investigate the fluorination influence on the photovoltaic performance of small molecular based organic solar cells (OSCs), six small molecules based on 2,1,3-benzothiadiazole (BT), and diketopyrrolopyrrole (DPP) as core and fluorinated phenyl (DFP) and triphenyl amine (TPA) as different terminal units (DFP-BT-DFP, DFP-BT-TPA, TPA-BT-TPA, DFP-DPP-DFP, DFP-DPP-TPA, and TPA-DPP-TPA) were synthesized. With one or two fluorinated phenyl as the end group(s), HOMO level of BT and DPP based small molecular donors were gradually decreased, inducing high open circuit voltage for fluorinated phenyl based OSCs. DFP-BT-TPA and DFP-DPP-TPA based blend films both displayed stronger nano-scale aggregation in comparison to TPA-BT-TPA and TPA-DPP-TPA, respectively, which would also lead to higher hole motilities in devices. Ultimately, improved power conversion efficiency (PCE) of 2.17% and 1.22% was acquired for DFP-BT-TPA and DFP-DPP-TPA based devices, respectively. These results demonstrated that the nano-scale aggregation size of small molecules in photovoltaic devices could be significantly enhanced by introducing a fluorine atom at the donor unit of small molecules, which will provide understanding about the relationship of chemical structure and nano-scale phase separation in OSCs.

## 1. Introduction

Recently, organic solar cells (OSCs) have received great attention due to their advantages of solution processability, light weight, low cost, and flexibility [1,2,3,4]. Generally, bulk-heterojunction architecture was adopted with the electron-efficient conjugated polymer or small molecule as the donor and electron-deficient fullerene derivative such as (6,6)-phenyl-C_71_-butyric acid methyl ester (PC_71_BM) as the acceptor [5,6,7]. In comparison to widely investigated polymeric counterparts, small molecule-based OSCs have distinct advantages of well-defined chemical structures, easy purification, and high purity without batch-to-batch variation [5,6,8,9]. Therefore, small molecule-based materials are more suitable for mass production compared to polymer based ones. Driven by the developing of high efficiency small molecular donors and the investigation of nano-scale phase separation at donor/acceptor interfaces, power conversion efficiency (PCE) of nearly 10% for small molecule-based single-junction OSCs has been achieved [10,11].

Due to the strong electron-withdrawing capability, the introducing of fluorine atoms onto the conjugated backbones of small molecules could reduce the HOMO energy levels, resulting in higher open circuit voltage (*V*_oc_) in OSCs [12,13,14,15,16,17,18,19]. Furthermore, the intra-molecular interaction of F–S and F–H could also induce closed packing properties and lead to superior hole mobiles [17,20,21,22]. Fluorinated molecules also showed good thermal and electrochemical stability, which would be helpful in the future commercial application. Although fluorinated small molecules have been broadly studied, fluorine atoms were mostly introduced to the acceptor unit of donor materials [18,19,23,24,25], works focus on the investigation of small molecules with a fluorine atom at the donor unit were still rare. Triphenylamine (TPA) based small molecules were common donor materials in organic semiconductor devices [8,26,27,28,29,30]. Nevertheless, the inferior planarity of TPA unit would induce weak intramolecular packing of small molecules and lead to a relatively low hole mobility in OSCs [31,32,33]. Conjugating a fluorine unit could therefore enhance the aggregation of TPA based small molecules and result in an appropriate nano-scale phase separation when blended with PC_71_BM. As a result, higher hole motilities and PCE were expected.

**Chart 1:**
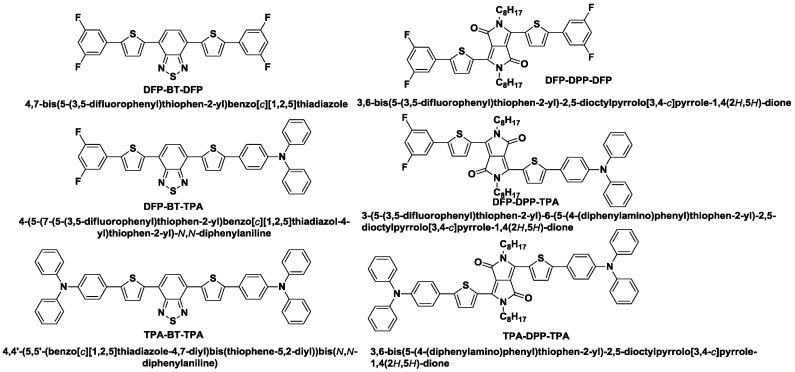
Chemical structures of small molecules (DFP-BT-DFP, DFP-BT-TPA, TPA-BT-TPA, DFP-DPP-DFP, DFP-DPP-TPA, and TPA-DPP-TPA). DFP: fluorinated phenyl; BT: 2,1,3-benzothiadiazole; DPP: diketopyrrolopyrrole; TPA: triphenyl amine.

In this contribution, six small molecules (DFP-BT-DFP, DFP-BT-TPA, TPA-BT-TPA, DFP-DPP-DFP, DFP-DPP-TPA, and TPA-DPP-TPA) based on 2,1,3-benzothiadiazole (BT) or diketopyrrolopyrrole (DPP) (Chart 1) as the core and TPA or fluorinated phenyl (DFP) groups as the flanks were synthesized and applied as the donors in OSCs [34,35,36]. BT or DPP groups were chosen as the acceptor units due to their uniquely planarity and remarkably electron-withdrawing capabilities, and various DFP groups were conjugated as the end groups to investigate the influence of fluorinated donor unit on the performance of small molecule based OSCs. With one or two DFP as the end group(s), the HOMO level of BT-based small molecular donors TPA-BT-TPA, DFP-BT-TPA, and DFP-BT-DFP were gradually decreased; DPP-based small molecular donors also exhibited similar tendency, which would be beneficial to achieve high *V*_oc_ in OSCs. Due to the inferior solubility of DFP-BT-DFP and DFP-DPP-DFP, only TPA-BT-TPA, TPA-DPP-TPA, DFP-BT-TPA, and DFP-DPP-TPA were used as donor materials in OSCs. DFP-BT-TPA and DFP-DPP-TPA based blend films both displayed stronger nano-scale aggregation in comparison to TPA-BT-TPA and TPA-DPP-TPA, respectively, leading to higher hole motilities in devices. Ultimately, a PCE of 2.17% with a *V*_oc_ of 0.90 V and a PCE of 1.22% with a *V*_oc_ of 0.78 V was acquired for DFP-BT-TPA and DFP-DPP-TPA-based devices, respectively. Our results demonstrated that the nano-scale aggregation size of small molecules in photovoltaic devices could be significantly enhanced by introducing a fluorine atom at the donor unit of small molecules, which provided useful information in the further design of high efficiency small molecular donors for OSCs.

## 2. Results and discussions

### 2.1. Synthesis

**Scheme 1:**
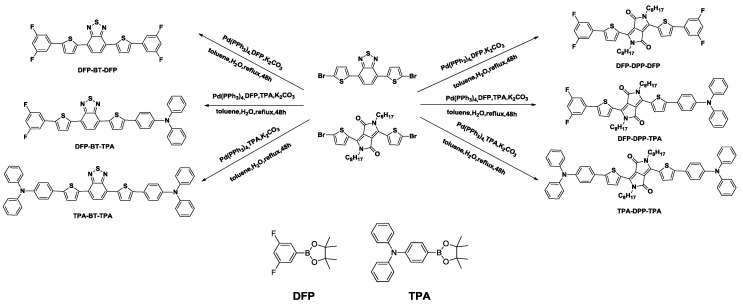
Synthetic route of six small molecules (DFP-BT-DFP, DFP-BT-TPA, TPA-BT-TPA, DFP-DPP-DFP, DFP-DPP-TPA, and TPA-DPP-TPA).

The synthesis route of six small molecules (DFP-BT-DFP, DFP-BT-TPA, TPA-BT-TPA, DFP-DPP-DFP, DFP-DPP-TPA, and TPA-DPP-TPA) is outlined in Scheme 1. All small molecules were synthesized by using the Suzuki cross-coupling reaction between a boronic acid ester and a brominated aromatic compound in toluene and K_2_CO_3_ aqueous solution under N_2_ with Pd(PPh_3_)_4_ as the catalyst procedure. DFP-BT-TPA, TPA-BT-TPA, DFP-DPP-TPA, and TPA-DPP-TPA show good solubility in common organic solvents such as chloroform (CHCl_3_), tetrahydrofuran (THF), and chlorobenzene (CB). However, DFP-BT-DFP and DFP-DPP-DFP showed poor solubility in these solvents, which might be ascribed to the fluorinated and symmetric molecular structure. As shown in Figure S1, DSC images demonstrated that all these small molecules were crystalline, which might be beneficial for the closed packing properties when blended with PC_71_BM. As shown in Figure S2 and Table 1, DFP-BT-DFP, DFP-BT-TPA, TPA-BT-TPA, DFP-DPP-DFP, DFP-DPP-TPA, and TPA-DPP-TPA all exhibited good thermal stability with a 5% weight loss of 350, 450, 461, 374, 400, and 408 °C, respectively. Our results revealed TPA-based small molecules possess better thermal stability than DFP based small molecules, which is beyond our expectation. The 5% weight loss temperature of DFP-BT-TPA and TPA-BT-TPA could reach to around 450 °C, making them very stable in the application of OSCs.

### 2.2. Optical Properties

**Figure 1:**
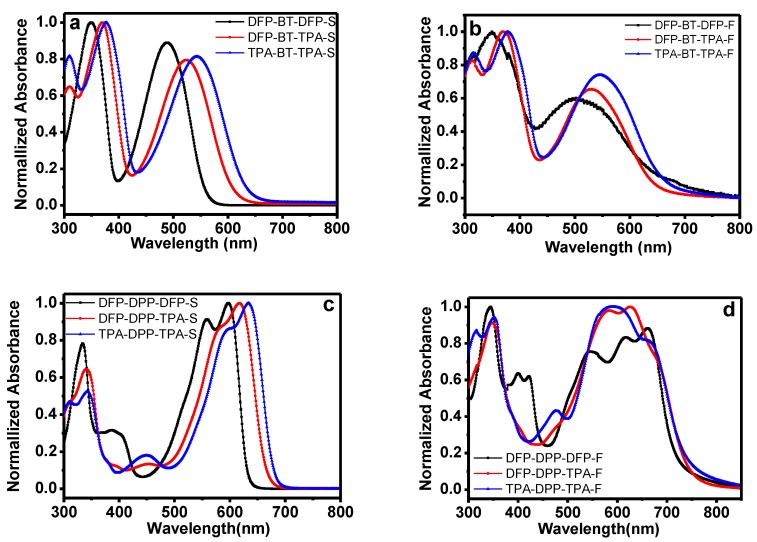
Normalized ultraviolet-visible spectroscopy (UV-VIS) absorption spectra of the six small molecules. (**a**,**c**) in dilute chlorobenzene (CB) solution; (**b**,**d**) as thin film on a quartz substrate.

The normalized ultraviolet-visible spectroscopy (UV-VIS) absorption spectra of six small molecules in dilute CB solution and as thin films at 25 °C are shown in Figure 1. These small molecules exhibit a broad absorption in the range from 300 to 700 nm with two distinguishable absorption bands, which could be attributed to the π-π* transition and the internal charge transfer (ICT) interaction between the donor and the acceptor units [4,21,28]. Upon going from solution to the solid state, the absorption spectra become broader and redshift 17 nm for DFP-BT-DFP, 5 nm for DFP-BT-TPA, and 5 nm for TPA-BT-TPA, respectively, which can be attributed to the closer π-π stacking in solid state. The onsets of film absorption spectra are 636, 643, and 659 nm, respectively, for these small molecules, and optical band gaps (*E*_g, opt_) were calculated to be 1.95, 1.93, and 1.88 eV, respectively. Similar to BT based small molecules, DFP-DPP-DFP, DFP-DPP-TPA, TPA-DPP-TPA show broader absorption over the range from 300 to 750 nm with two distinguishable absorption bands. Specifically, the absorption for DFP-DPP-DFP showed stronger shoulder peaks, revealing more ordered molecular stacking capabilities in the solid state [37,38]. From the absorption onsets, the optical band gaps were estimated to be 1.73, 1.66, and 1.63 eV for DFP-DPP-DFP, DFP-DPP-TPA, and TAP-DPP-TPA, respectively.

### 2.3. Cyclic Voltammetry

**Figure 2:**
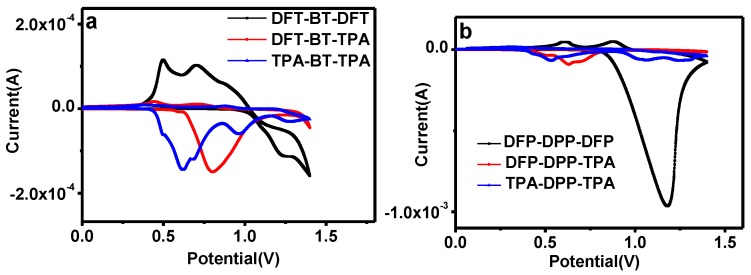
Cyclic voltammograms of small molecules. (**a**) DFP-BT-DFP, DFP-BT-TPA, TPA-BT-TPA; and (**b**) DFP-DPP-DFP, DFP-DPP-TPA, TPA-DPP-TPA.

Cyclic voltammetry (CV) experiments were carried out to evaluate electrochemical characteristics of these small molecules. As shown in Figure 2, the onset oxidation potentials (*E*_ox_) were 1.02, 0.63, 0.46 V for DFP-BT-DFP, DFP-BT-TPA, and TPA-BT-TPA, respectively, According to the equation *E*_HOMO_ = −(4.71 + *E*_ox_) (eV), highest occupied molecular orbital (HOMO) energy levels of these small molecules could be determined to be −5.73, −5.34, and −5.17 eV. With the combination of the optical band gap and the equation *E*_LUMO_ = *E*_HOMO_ + *E*_g, opt_, [39] the lowest unoccupied molecular orbital (LUMO) energy levels were calculated to be −3.78, −3.41, and −3.29 eV for DFP-BT-DFP, DFP-BT-TPA, and TPA-BT-TPA, respectively. Similarly, the HOMO and LUMO energy levels were determined to be −5.59, −5.13, −5.07 and −3.86, −3.47, −3.44 eV for DFP-DPP-DFP, DFP-DPP-TPA, and TAP-DPP-TPA, respectively. These results demonstrated that the incorporation of fluorine atom could significantly decrease the HOMO energy levels of small molecules, which would be beneficial to achieve high *V*_oc_ in OSCs. The data are also summarized in Table 1.

### 2.4. Photovoltaic Properties

**Figure 3:**
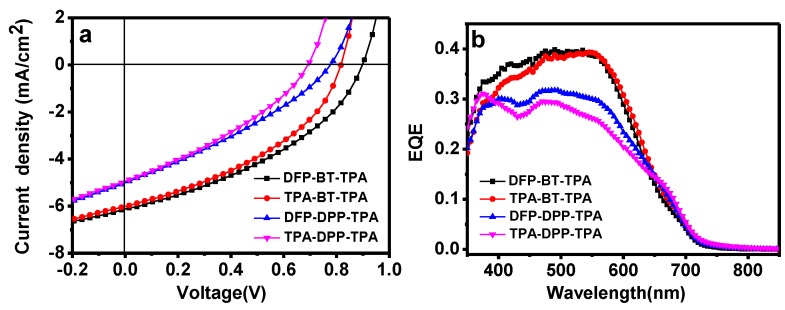
*J*-*V* characteristics (**a**) and external quantum efficiencie (EQE) curves (**b**) of devices fabricated from the blend of small molecule: PC_71_BM.

Photovoltaic properties of these small molecules were investigated by a general device structure of indium tin oxides (ITO)/poly(3,4-ethylenedioxythiophene):poly(styrenesulfonate) (PEDOT:PSS)/donor:PC_71_BM/LiF/Al. Different spin-coating speeds and weight ratios of small molecules to PC_71_BM in a CB solution were screened to optimize the photovoltaic performance. Due to the inferior solubility of DFP-BT-DFP and DFP-DPP-DFP, typical cluster structures with boosted big domains could be easily observed by naked eyes. As a result, photovoltaic performance of these two small molecules based devices would not be discussed. For other small molecules, the current density-voltage (*J*-*V*) curves are shown in Figure 3a and device characteristics are summarized in Table 2. After optimization, superior PCE values could be achieved with a weight ratio of 1:4 (*w*/*w*) for all devices, and a PCE of 1.95% with a *V*_oc_ of 0.82 V, *J*_sc_ of 5.97 mA·cm^−2^ and fill factor (FF) of 0.40 was achieved for TPA-BT-TPA devices. When one TPA unit was replaced by one DFP group, increased *V*_oc_ of 0.90 V and *J*_sc_ of 6.12 mA·cm^−2^ were obtained for DFP-BT-TPA based device, leading to higher PCE of 2.17%. Similarly, DFP-DPP-TPA based solar cells exhibited a higher PCE of 1.22% with a *V*_oc_ of 0.78 V than that of TPA-DPP-TPA based devices. This improvement could be attributed to the increase of *V*_oc_ and *J*_sc_. Since *V*_oc_ is related to the offset between HOMO level of donor materials and LUMO level of PC_71_BM, the decrease of HOMO levels for DFP based small molecules would lead to higher *V*_oc_ [3]. Meanwhile, DFP based small molecules also possesses bigger nano-scale aggregates, and resulting higher hole mobilities in the active layer (*vide infra*), which would also lead to higher *J*_sc_ in devices.

To verify the *J*_sc_ obtained from *J*-*V* measurement, the external quantum efficiencies (EQEs) of devices were measured. As shown in Figure 3b, the EQE images of devices all displayed a broad response in the range from 350 to 600 nm, and the maximum EQE value of DFP-BT-TPA and TPA-BT-TPA-based devices are both 40%, which are higher than that of DFP-DPP-TPA and TPA-DPP-TPA. *J*_sc_ values integrated from EQE curves all agreed approximately with that obtained from *J*-*V* measurement.

The hole mobilities of these devices were measured by the space charge limited current (SCLC) method. Hole-only devices were fabricated with a structure of ITO/PEDOT:PSS/donor:PC_71_BM/Au and resulting hole mobility value (μ) was calculated from the dark *J*-*V* experiments. Dark *J*-*V* curves were fitted by using the Mott–Gurney equation: *J* = 9ε_o_ε_r_μ*V*^2^/8L^3^, where J is the space charge limited current, ε_o_ is the vacuum permittivity, ε_r_ is the permittivity of the active layer, μ is the hole mobility of small molecules, and L is the thickness of the active layer. μ of devices based on DFP-BT-TPA and DFP-DPP-TPA were evaluated to be 3.81 × 10^−4^ and 9.35 × 10^−5^ cm^2^·V^−1^·s^−1^, respectively, and μ of TPA-BT-TPA and TPA-DPP-TPA based devices were determined to be 4.42 × 10^−5^ and 3.26 × 10^−5^ cm^2^·V^−1^·s^−1^, respectively. μ of DFP based devices were obviously higher than that of TPA based ones, which might be attributed to their planar chemical structure and resulting bigger nano-scale aggregation in the blend films.

### 2.5. Film Morphology

**Figure 4:**
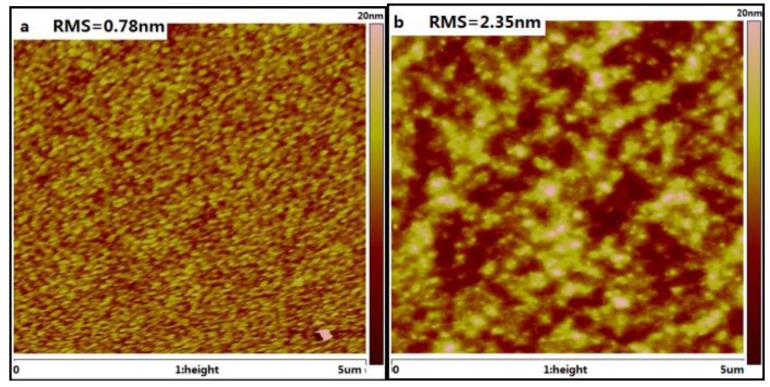

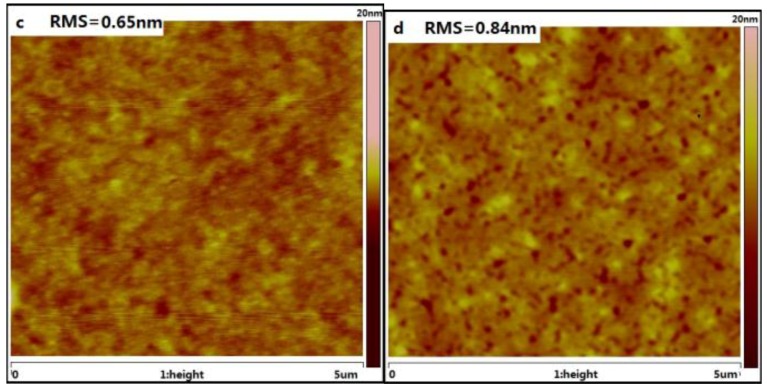
Atomic force microscopy (AFM) (5 × 5 μm^2^) height images of blend films. (**a**) TPA-BT-TPA:PC_71_BM; (**b**) DFP-BT-TPA:PC_71_BM; (**c**) TPA-DPP-TPA:PC_71_BM; and (**d**) DFP-DPP-TPA:PC_71_BM.

Atomic force microscopy (AFM) experiments were carried out to investigate the surface morphology of the active layer. Well-mixed blend films were in favor of exciton dissociation in devices, whereas appropriate nano-scale aggregation of small molecules was also important for the charge transport. Therefore, suitable nano-scale phase separation is critical to obtain a superior photovoltaic performance [40,41]. As shown in Figure 4, blend films based on TPA-BT-TPA and TPA-DPP-TPA were both uniform and smooth with a root-mean-square (RMS) roughness value of 0.78 and 0.65 nm, which might be ascribed to the inferior packing properties of TPA group. For devices based on DFP-BT-TPA and DFP-DPP-TPA, rougher blend film surface with a RMS roughness value of 2.35 nm and 0.84 nm were acquired, demonstrating the domain size of DFP-BT-TPA and DFP-DPP-TPA were higher than TPA-BT-TPA and TPA-DPP-TPA in devices. Our results demonstrated that incorporating a DFT group as the end groups could enhance the nano-scale aggregation in the devices, which would be beneficial to the charge transport in the blend films, and lead to higher *J*_sc_ and PCE.

## 3. Experimental Section

### 3.1. Materials

All chemicals were purchased from commercial sources and used without further purification, and all solvents were purified by standard techniques. Additionally, all reactions were carried out under the nitrogen atmosphere unless otherwise stated. The catalyst procedure Pd(PPh_3_)_4_ [34], 4,7-bis(5-bromothiophen-2-yl)benzo[c][1,2,5]thiadiazole [35] and 3,6-bis(5-bromothiophen-2-yl)-2,5- dioctylpyrrolo[3,4-c]pyrrole-1,4-dione [36] were synthesized according to the literature procedures.

### 3.2. Measurements and Characterization

All reactions were monitored by thin layer chromatography (TLC) on silica gel 60 F254 (Merck, Beijing, China, 0.2 mm), and column chromatography was performed on silica gel (200~300 mesh). ^1^H and ^13^C NMR spectra were recorded on a Bruker AV 400 spectrometer (Saarbrücken, Germany) using CDCl_3_ as the solvents. Differential scanning calorimetry (DSC) measurements were performed on the Perkin-Elmer Diamond DSC instrument (Shanghai, China) under a nitrogen atmosphere with a heating rate of 20 °C/min. UV-VIS absorption spectra was recorded on the PerkinElmer UV-VIS spectrometer model Lambda 750 (Shanghai, China). The electrochemical behavior of small molecules was achieved using CHI 630A electrochemical analyzer (Beijing, China) with a standard three-electrode electrochemical cell in 0.1 M Bu_4_NPF_6_ in CH_3_CN solution. A glassy carbon working electrode, a Pt wire counter electrode, and an Ag/AgNO_3_ (0.01 M in CH_3_CN) reference electrode were used. Elemental analyses were performed on a Flash EA 1112 analyzer (Lakewood, CO, USA), and atomic force microscopy (AFM) measurements were carried out in the tapping mode using a Digital Instrument Multimode Nanoscope IIIA (Plainview, TX, USA). The thickness of the active layers was determined by the Dektak 6 M surface profilometer (Beijing, China).

### 3.3. Fabrication and Characterization of SCLC

Devices used to measure the space charge limited current (SCLC) were fabricated with a configuration of ITO/PEDOT:PSS/small molecule:PC_71_BM/Au. The conductivity of ITO was 20 Ω. PEDOT:PSS is Baytron Al 4083 from H.C.Starck (Leverkusen, Germany) and filtered with a 0.45 μm polyethersulfone (PES) film before use. The PEDOT:PSS was spin-coated on top of a ITO substrate from a speed of 3000 rpm/s for 60 s and dried at 130 °C for 15 min on a hotplate. The thickness of the PEDOT:PSS layer was about 40 nm. Small molecule and PC_71_BM was dissolved in chlorobenzene (CB) at 110 °C overnight, and then spin-coated onto the PEDOT:PSS layer. The top electrode was subsequently thermally evaporated with a 100 nm of gold under a pressure of 10^−4^ Pa by a shadow mask. Dark current-voltage characteristics were recorded using an Agilent B2902A Source (Taiwan) under darkness in a range of 0 V to 5.0 V.

### 3.4. Fabrication and Characterization of PSCs

PSCs were fabricated with the device configuration of ITO/PEDOT:PSS/small molecule:PC_71_BM/LiF/Al. ITO glasses with a conductivity of 20 Ω were cleaned before use. PEDOT:PSS is Baytron Al 4083 from H.C.Starck, and filtered with a 0.45 µm PES film before use. The thin layer of PEDOT:PSS was spin-coated on top of a cleaned ITO substrate at 3000 rpm/s for 60 s and dried subsequently at 130 °C for 15 min on a hotplate. The thickness of the PEDOT:PSS layer was about 40 nm. The active layer was subsequently prepared by spin-coating the blend solution of small molecule and PC_71_BM on the top of ITO/PEDOT:PSS. The top electrode was thermally evaporated with a 0.6 nm LiF layer, and followed by 100 nm of aluminum under a pressure of 10^−4^ Pa through a shadow mask. Five cells were fabricated on one substrate with an effective area of 0.04 cm^2^. The current-voltage (*I*-*V*) curves of devices were completed by a computer-controlled Keithley 236 Source Measure Unit. An AM 1.5G AAA class solar simulator (model XES-301S, SAN-EI) in an intensity of 100 mW/cm^2^ was used as the simulative sun light source, and the light intensity of the light was calibrated under a standard single-crystal Si photovoltaic cell.

### 3.5. General Procedure for the Synthesis of Small Molecules

The general procedure for the synthesis were shown in Scheme 1, A solution of BT or DPP, DFP or TPA, K_2_CO_3_, toluene (50 mL), and H_2_O (5 mL) was carefully gas and degassed before and after Pd(PPh_3_)_4_ was added, and the mixture was stirring at 100 °C for two days. After the reaction, the mixture was poured into water (100 mL) and extracted with CHCl_3_. The organic layer was washed with water three times, and then dried by MgSO_4_. After evaporating to dryness, the pure product was obtained from column chromatography by a silica gel.

4,7-bis(5-(3,5-difluorophenyl)thiophen-2-yl)benzo[c][1,2,5]thiadiazole (DFP-BT-DFP): BT (0.96 g, 2.1 mmol), DFP (1.01 g, 4.2 mmol), K_2_CO_3_ (1.38 g, 10.0 mmol), and Pd(PPh_3_)_4_ (127.1 mg, 0.11 mmol) were used. Yield: 0.73 g, 66% purple crystal. ^1^H NMR (400 MHz, CDCl_3_) δ. 8.12 (d, 1H), 7.94 (s, 1H), 7.44 (d, 1H) 7.52 (m, 2H), 6.77 (m, 1H). ^13^C NMR (100 MHz, CDCl_3_) δ. 155.61, 151.55, 148.01, 143.27, 142.00, 137.55, 131.73, 120.63, 116.91. Analyze (Anal.) Calcd for C_26_H_12_F_4_N_2_S_3_: C, 59.53; H, 2.31; N, 5.34; Found: C, 59.42; H, 2.40; N, 5.26. MALDI-TOF: calculated for C_26_H_12_F_4_N_2_S_3_ 524.6, found 523.8.

4-(5-(7-(5-(3,5-difluorophenyl)thiophen-2-yl)benzo[c][1,2,5]thiadiazol-4-yl)thiophen-2-yl)-*N*,*N*-diphenylaniline (DFP-BT-TPA): BT (1.15 g, 2.5 mmol), DFP (0.60 g, 2.5 mmol), TPA (0.93 g, 2.5 mmol), K_2_CO_3_ (1.38 g, 10.0 mmol), and Pd(PPh_3_)_4_ (144.3 mg, 0.11 mmol) were used. Yield: 0.67 g, 41% purple crystal. ^1^H NMR (400 MHz, CDCl_3_) δ. 8.14 (d, 1H), 8.08 (d, 1H), 7.90 (t, 2H) 7.57 (d, 2H), 7.43 (d, 1H), 7.34 (d, 1H), 7.29 (t, 3H), 7.22 (d, 2H), 7.16–7.00 (m, 8H), 6.75 (t, 1H). ^13^C NMR (100 MHz, CDCl_3_) δ. 161.66, 158.56, 153.96, 145.33, 143.04, 139.03, 132.72, 129.49, 127.99, 124.80, 123.41, 123.32, 115.51. Anal. Calcd for C_38_H_23_F_2_N_3_S_3_: C, 69.60; H, 3.54; N, 6.41 Found: C, 69.45; H, 3.52; N, 6.40. MALDI-TOF: calculated for C_38_H_23_F_2_N_3_S_3_ 655.8, found 654.9.

4,4′-(5,5′-(benzo[c][1,2,5]thiadiazole-4,7-diyl)bis(thiophene-5,2-diyl))bis(*N*,*N*-diphenylaniline) (TPA-BT-TPA): BT (1.01 g, 2.2 mmol), TPA (1.63 g, 4.4 mmol), K_2_CO_3_ (1.22 g, 8.8 mmol), and Pd(PPh_3_)_4_ (127.1 mg, 0.11 mmol) were used. Yield: 1.30 g, 75% purple crystal. ^1^H NMR (400 MHz, CDCl_3_) δ. 8.11 (s, 2H), 7.85 (s, 2H), 7.56 (m, 4H), 7.29 (m, 10H), 7.08 (m, 16H). ^13^C NMR (100 MHz, CDCl_3_) δ. 152.57, 147.58, 147.39, 129.35, 128.67, 128.03, 126.60, 125.61, 125.14, 124.64, 123.46, 123.23. Anal. Calcd for C_50_H_34_N_4_S_3_: C, 76.30; H, 4.35; N, 7.12. Found: C, 76.17; H, 4.36; N, 7.02. MALDI-TOF: calculated for C_50_H_34_N_4_S_3_ 787.0, found 785.9.

3,6-bis(5-(3,5-difluorophenyl)thiophen-2-yl)-2,5-dioctylpyrrolo[3,4-c]pyrrole-1,4(2H,5H)-dione(DFP-DPP-DFP): DPP (1.37 g, 2.0 mmol) and DFP (0.96 g, 4.0 mmol), K_2_CO_3_ (1.11 g, 8.0 mmol), and Pd(PPh_3_)_4_ (115.5 mg, 0.11 mmol) were used. Yield: 0.91 g, 66% gray solid. ^1^H NMR (400 MHz, CDCl_3_) δ. 8.94 (d, 2H), 7.48 (d, 2H), 7.19 (d, 4H), 6.82 (t, 2H), 4.11 (t, 4H), 1.77 (t, 4H), 1.46–1.28 (m, 20H), 0.86 (t, 6H) ^13^C NMR (100 MHz, CDCl_3_) δ. 161.26, 155.77, 155.15, 149.36, 146.58, 139.32, 136.46, 129.90, 125.89, 109.15, 108.85, 42.32, 31.79, 30.01, 29.28, 29.21, 26.90, 22.63, 14.07. Anal. Calcd for C_42_H_44_F_4_N_2_O_2_S_2_: C, 67.36; H, 5.92; N, 3.74. Found: C, 67.12; H, 5.92; N, 3.73. MALDI-TOF: calculated for C_42_H_44_F_4_N_2_O_2_S_2_ 748.9, found 748.1.

3-(5-(3,5-difluorophenyl)thiophen-2-yl)-6-(5-(4-(diphenylamino)phenyl)thiophen-2-yl)-2,5-dioctylpyrrolo[3,4-c]pyrrole-1,4(2H,5H)-dione(DFP-DPP-TPA): DPP (1.91 g, 2.8 mmol), DFP (0.67 g, 2.8 mmol) and TPA (1.04 g, 2.8 mmol), K_2_CO_3_ (3.33 g, 14.0 mmol), and Pd(PPh_3_)_4_ (161.7 mg, 0.14 mmol) were used. Yield: 0.76 g, 31% gray solid. ^1^H NMR (400 MHz, CDCl_3_) δ. 9.04 (s, 1H), 8.87 (s, 1H), 7.52 (s, 2H), 7.44 (s, 1H), 7.36 (s, 1H), 7.30 (t, 4H), 7.14 (d, 6H), 7.08 (dd, 4H), 6.78 (s, 1H), 4.09 (t, 4H), 1.76 (t, 4H), 1.45–1.26 (m, 20H), 0.85 (t, 6H). ^13^C NMR (100 MHz, CDCl_3_) δ. 148.74, 147.02, 137.71, 129.47, 126.99, 126.30, 125.09, 123.80, 123.64, 122.58, 109.05, 108.77, 42.32, 31.82, 30.04, 29.27, 29.22, 26.94, 22.64, 14.12. Anal. Calcd for C_55_H_59_F_2_N_3_O_2_S_2_: C, 73.71; H, 6.64; N, 4.69. Found: C, 73.25; H, 6.35; N, 4.73. MALDI-TOF: calculated for C_55_H_59_F_2_N_3_O_2_S_2_ 896.2, found 895.3.

3,6-bis(5-(4-(diphenylamino)phenyl)thiophen-2-yl)-2,5-dioctylpyrrolo[3,4-c]pyrrole-1,4(2H,5H)-dione(TPA-DPP-TPA): DPP (1.30 g, 1.9 mmol) and TPA (1.41 g, 3.8 mmol), K_2_CO_3_ (1.31 g, 9.5 mmol), and Pd(PPh_3_)_4_ (115.5 mg, 0.10 mmol) were used. Yield: 1.35 g, 70% gray solid. ^1^H NMR (400 MHz, CDCl_3_) δ. 7.30 (t, 15H), 7.10 (d, 17H), 4.10 (t, 4H), 1.80 (t, 4H), 1.46–1.26 (m, 20H), 0.84 (t, 6H). ^13^C NMR (100 MHz, CDCl_3_) δ. 162.54, 160.20, 155.76, 148.66, 147.40, 132.11, 129.44, 126.93, 124.82, 123.60, 123.69, 122.72, 110.77, 42.28, 31.81, 30.03, 29.24, 29.22, 26.93, 22.63, 14.10. Anal. Calcd for C_66_H_66_N_4_O_2_S_2_: C, 78.38; H, 6.58; N, 5.54. Found: C, 78.17; H, 6.61; N, 5.38. MALDI-TOF: calculated for C_66_H_66_N_4_O_2_S_2_ 1011.4, found 1010.1.

## 4. Conclusions

In conclusion, six small molecules (DFP-BT-DFP, DFP-BT-TPA, TPA-BT-TPA, DFP-DPP-DFP, DFP-DPP-TPA, and TPA-DPP-TPA) based on 2,1,3-benzothiadiazole (BT) or diketopyrrolopyrrole (DPP) as the core and TPA or fluorinated phenyl (DFP) as the end groups were designed and synthesized as the donors in OSCs. To investigate the influence of fluorinated donor unit on the photovoltaic performance of devices, various DFP groups were conjugated. With one or two DFP as the end group(s), HOMO level of TPA-BT-TPA, DFP-BT-TPA, and DFP-BT-DFP was gradually decreased; similar tendency could also be observed for DPP based small molecular donors, inducing high *V*_oc_ for DFP based OSCs. DFP-BT-TPA and DFP-DPP-TPA based blend films both displayed stronger nano-scale aggregation in comparison to TPA-BT-TPA and TPA-DPP-TPA, respectively, which would also lead to higher hole motilities in devices. Ultimately, a PCE of 2.17% with a *V*_oc_ of 0.90 V was acquired for DFP-BT-TPA based devices. Our results demonstrated that the nano-scale aggregation size of small molecules in photovoltaic devices could be significantly enhanced by introducing a fluorine atom at the donor unit of small molecules. Although the PCE is not attractive enough, this work will provide understanding about the relationship of chemical structure and nano-scale phase separation in OSCs.

## Figures and Tables

**Table 1:** Optical, electrochemical, and physical properties of small molecules.

Donor	λ_abs_(S) (nm) ^a^	λ_abs_(F) (nm) ^b^	λ_onset_ (nm)	*E*_g, opt_(ev)	HOMO ^c^	LUMO ^d^	*T*_m_ (°C)	*T*_g_ (°C)
DFP-BT-DFP	488	505	636	1.95	−5.73	−3.78	246.6	350
DFP-BT-TPA	525	530	643	1.93	−5.34	−3.41	248.8	450
TPA-BT-TPA	544	547	659	1.88	−5.17	−3.29	254.4	461
DFP-DPP-DFP	598	616, 663	715	1.73	−5.59	−3.86	216.9	374
DFP-DPP-TPA	619	583, 627	746	1.66	−5.13	−3.47	195.3	400
TPA-DPP-TPA	634	588, 656	760	1.63	−5.07	−3.44	217.3	408

**^a^** S stands for in solutions; **^b^** F stands for as films; **^c^** HOMO means highest occupied molecular orbital; **^d^** LUMO means lowest unoccupied molecular orbital.

**Table 2:** Photovoltaic performance and hole mobilities of small molecule-based devices. FF: fill factor; PCE: power conversion efficiency; SCLC: space charge limited current.

Donor Molecule	*V*_oc_ (V)	*J*_sc_ (mA·cm^−2^)	FF	PCE (max/ave) ^a^ (%)	Thickness (nm)	SCLC (cm^2^·V^−1^·s^−1^)
DFP-BT-TPA	0.90	6.12	0.39	2.17/2.08	76	3.81 × 10^−4^
TPA-BT-TPA	0.82	5.97	0.40	1.95/1.85	82	4.42 × 10^−5^
DFP-DPP-TPA	0.78	4.95	0.32	1.22/1.17	70	9.35 × 10^−5^
TPA-DPP-TPA	0.70	4.90	0.34	1.15/1.02	85	3.26× 10^−5^

^a^ Average value recorded over 20 devices.

## Supplementary Materials

The following are available online at http://www.mdpi.com/2079-4991/6/4/80/s1.
