# Supplementary material for: The Influence of Fluorination on Nano-Scale Phase Separation and Photovoltaic Performance of Small Molecular/PC71BM Blends
Source: Nanomaterials (Basel). 2016 Apr 22;6(4):80. doi: 10.3390/nano6040080 (PMC5302554; doi:10.3390/nano6040080)
Supplement: Supplementary file 1 [file nanomaterials-06-00080-s001.pdf]

Supplementary Material: The Influence of Fluorination on Nano-Scale Phase Separation and Photovoltaic Performance of Small Molecular/PC71BM Blends

Zhen Lu, Wen Liu, Jingjing Li, Tao Fang, Wanning Li, Jicheng Zhang, Feng Feng and Wenhua Li

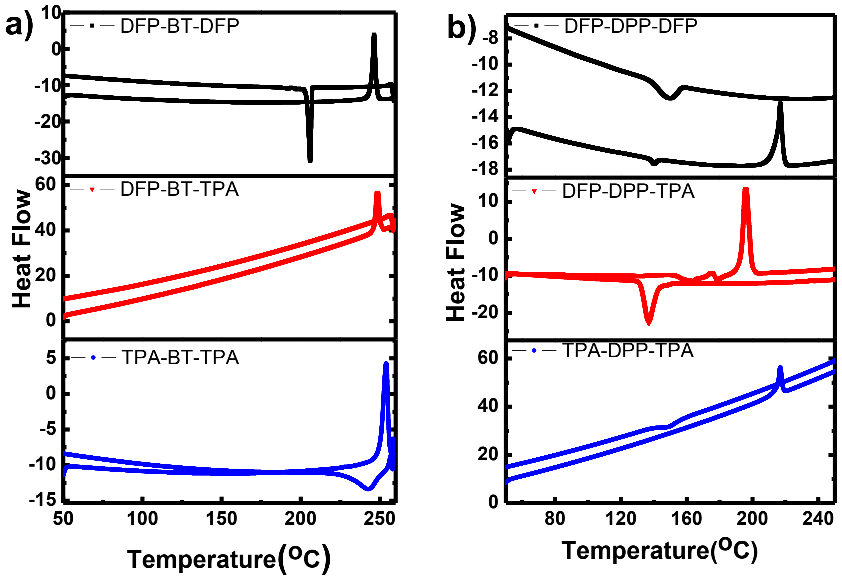

Figure S1. Differential scanning calorimetry (DSC) of small molecules (**a**) (DFP-BT-DFP, DFP-BT-TPA, TPA-BT-TPA); (**b**) (DFP-DPP-DFP, DFP-DPP-TPA, TPA-DPP-TPA) with a heating rate of 20 °C/min. DFP: fluorinated phenyl; BT: 2,1,3-benzothiadiazole; DPP: diketopyrrolopyrrole; TPA: triphenyl amine.

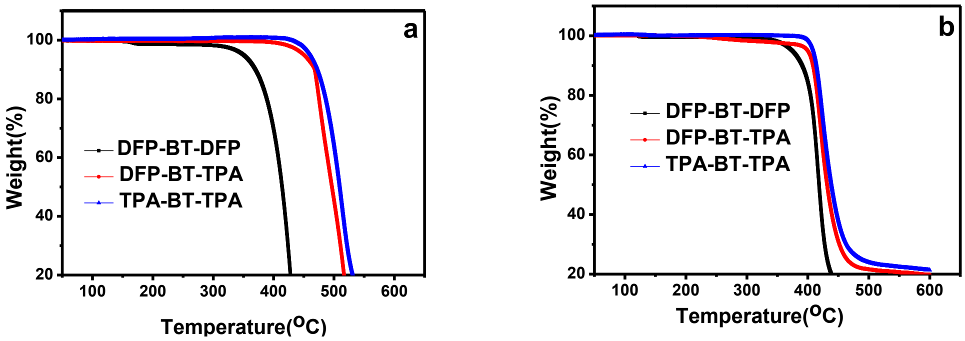

Figure S2. Thermal gravimetric analysis (TGA) curves of (**a**) DFP-BT-DFP, DFP-BT-TPA, TPA-BT-TPA and (**b**) DFP-DPP-DFP, DFP-DPP-TPA, TPA-DPP-TPA.

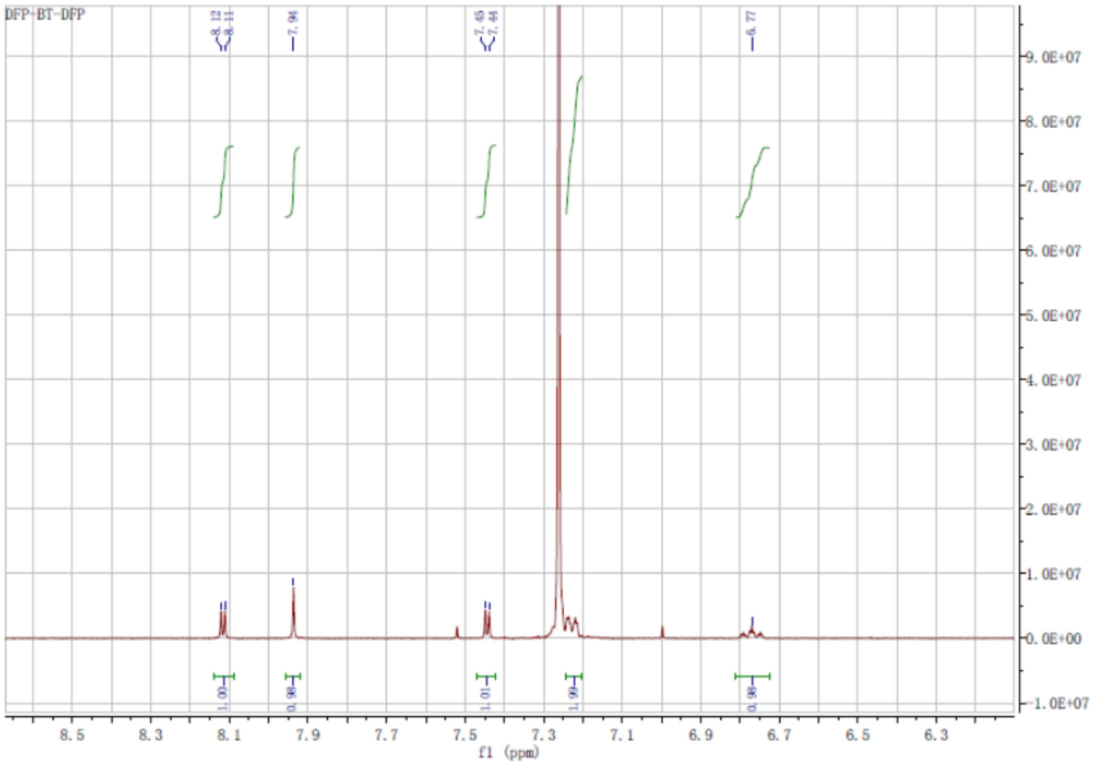

Figure S3. ^{1}H NMR spectrum of DFP-BT-DFP (measured in $CDCl_3$).

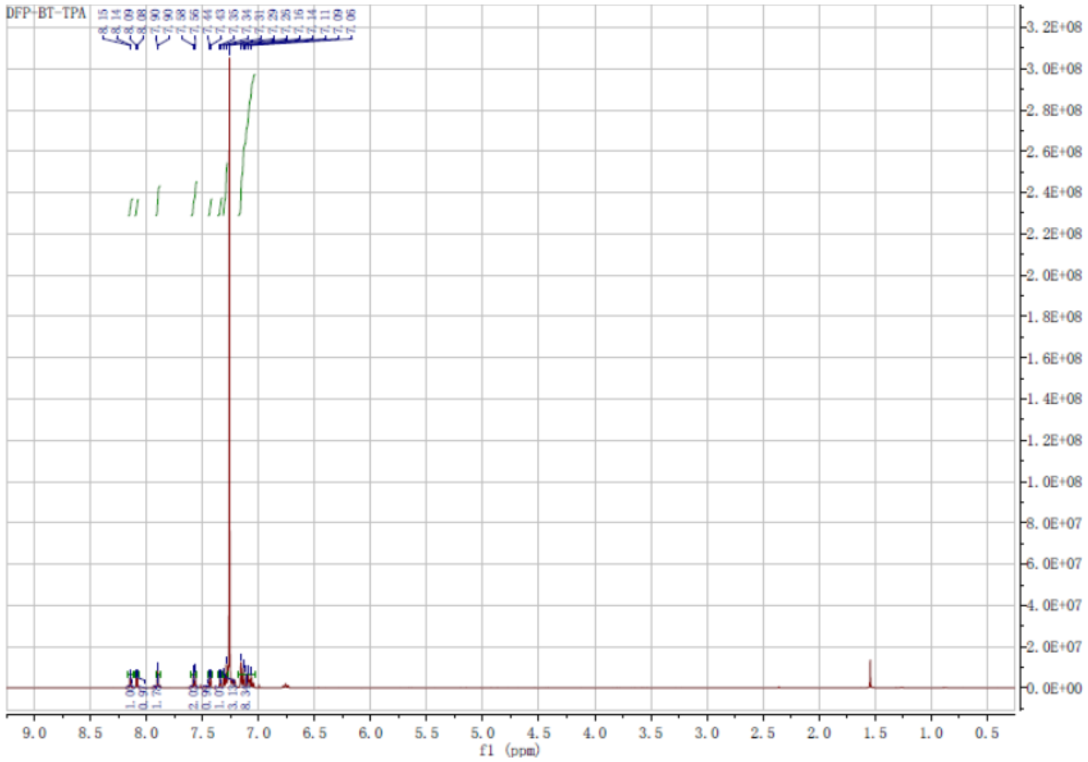

Figure S4. ^{1}H NMR spectrum of DFP-BT-TPA (measured in $CDCl_3$).

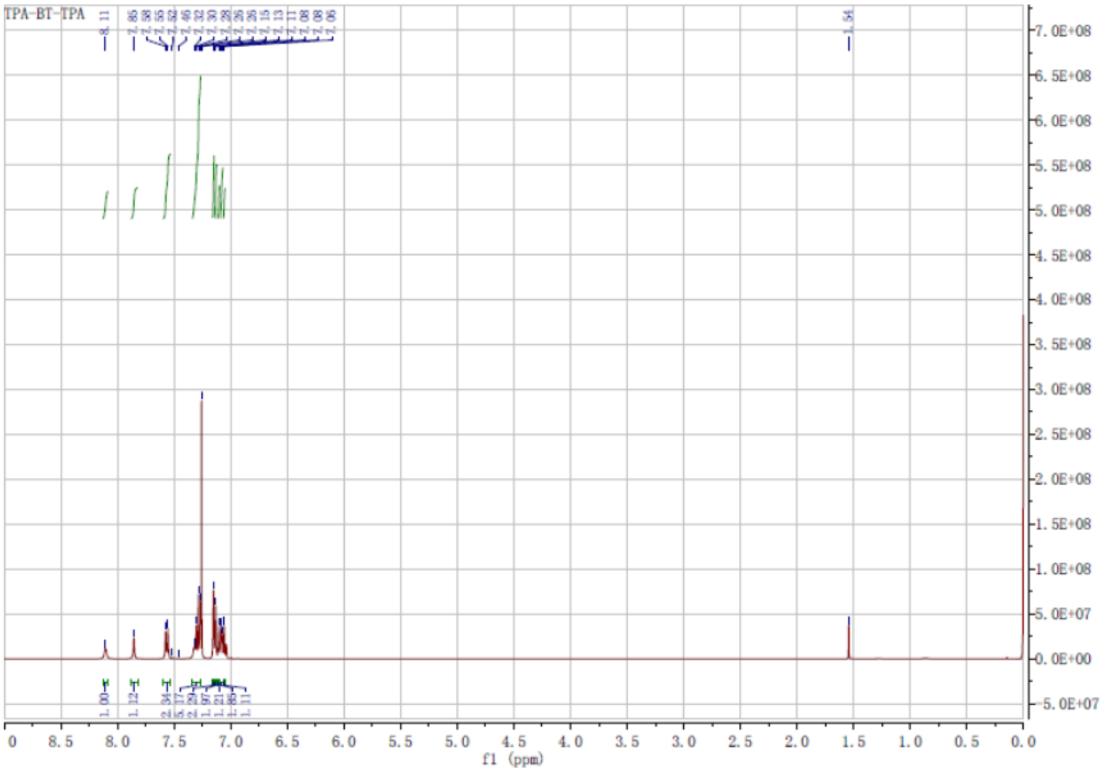

Figure S5. ^{1}H NMR spectrum of TPA-BT-TPA (measured in $CDCl_3$).

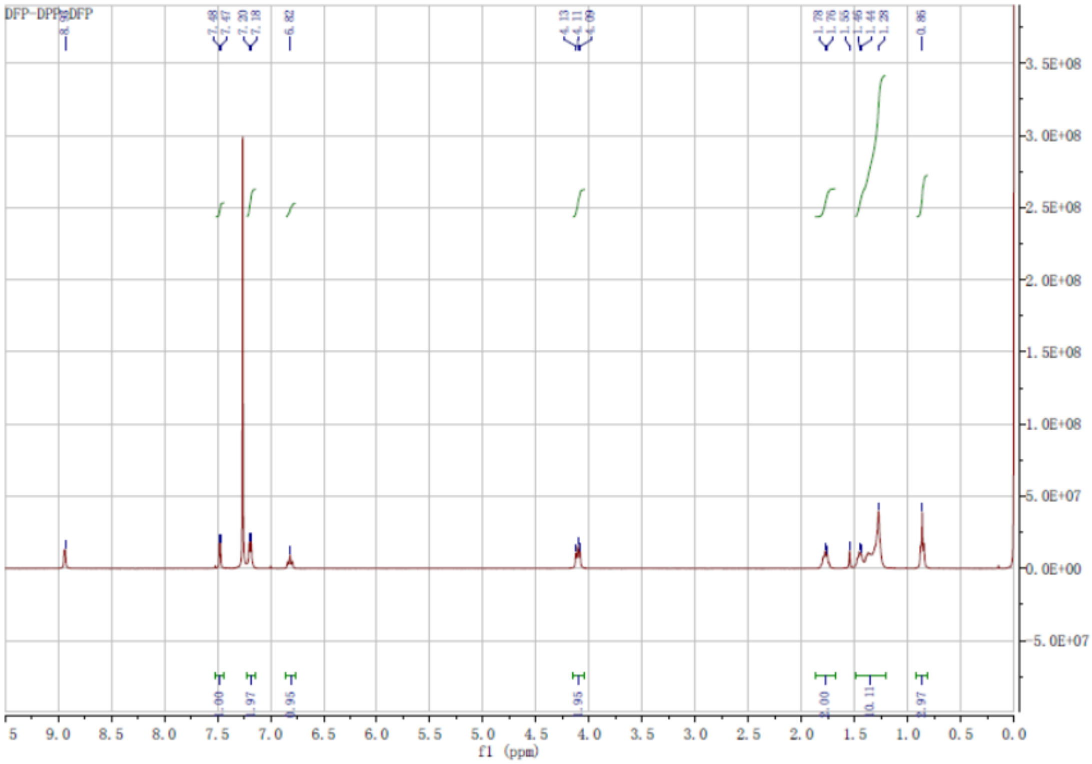

Figure S6. ^{1}H NMR spectrum of DFP-DPP-DFP (measured in $CDCl_3$).

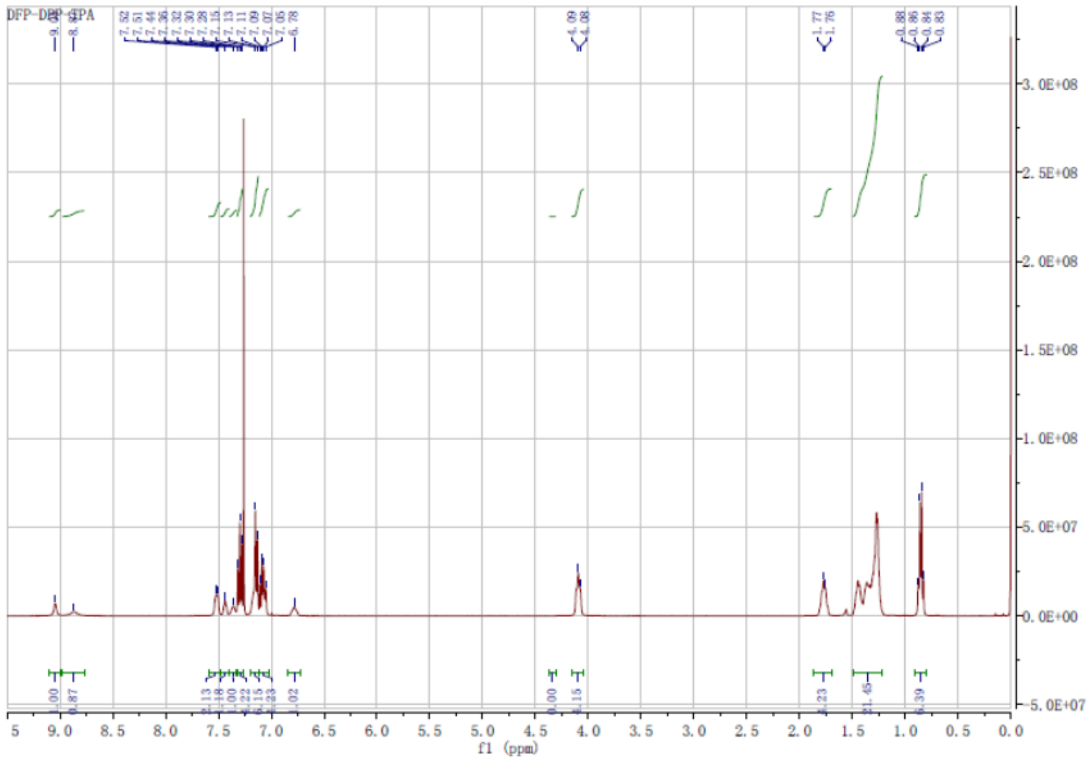

Figure S7. ^{1}H NMR spectrum of DFP-DPP-TPA (measured in $CDCl_3$).

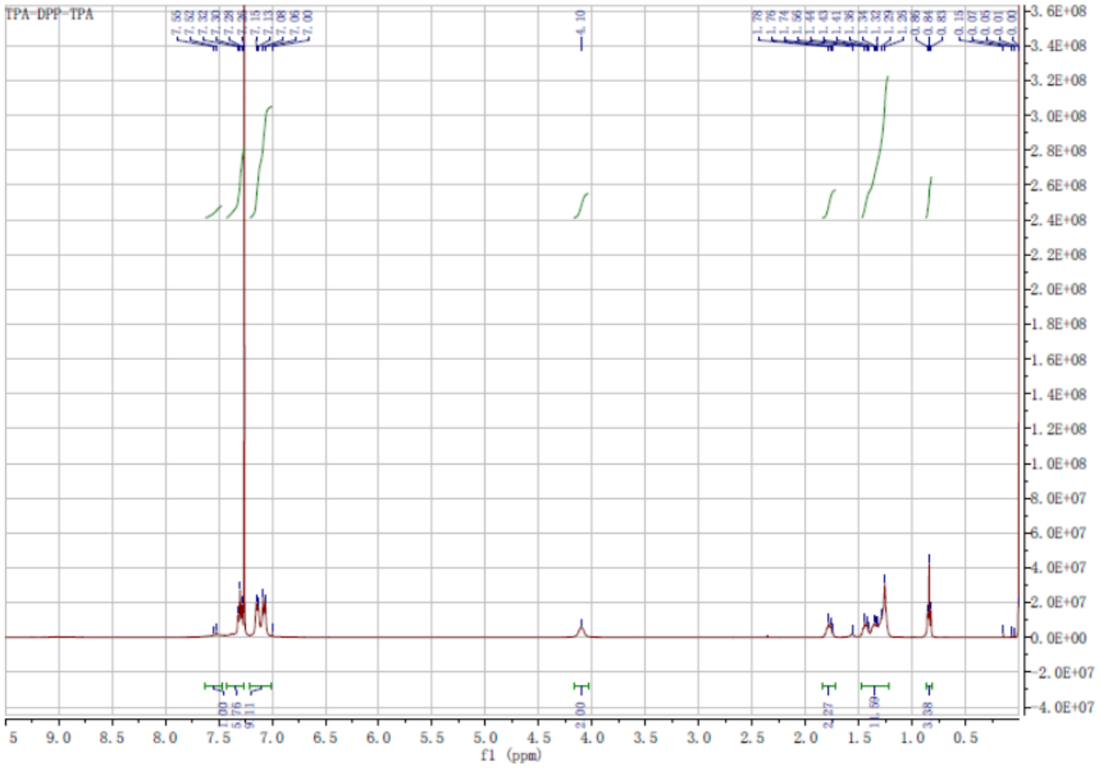

Figure S8. ^{1}H NMR spectrum of TPA-DPP-TPA (measured in $CDCl_3$).

© 2016 by the authors; licensee MDPI, Basel, Switzerland. This article is an open access article distributed under the terms and conditions of the Creative Commons by Attribution (CC-BY) license (http://creativecommons.org/licenses/by/4.0/).
